# Enhancing Camera Calibration for Traffic Surveillance with an Integrated Approach of Genetic Algorithm and Particle Swarm Optimization

**DOI:** 10.3390/s24051456

**Published:** 2024-02-23

**Authors:** Shenglin Li, Hwan-Sik Yoon

**Affiliations:** Department of Mechanical Engineering, The University of Alabama, Tuscaloosa, AL 35487, USA; sli90@crimson.ua.edu

**Keywords:** camera calibration, traffic surveillance, genetic algorithm, particle swarm optimization

## Abstract

Recent advancements in sensor technologies, coupled with signal processing and machine learning, have enabled real-time traffic control systems to effectively adapt to changing traffic conditions. Cameras, as sensors, offer a cost-effective means to determine the number, location, type, and speed of vehicles, aiding decision-making at traffic intersections. However, the effective use of cameras for traffic surveillance requires proper calibration. This paper proposes a new optimization-based method for camera calibration. In this approach, initial calibration parameters are established using the Direct Linear Transformation (DLT) method. Then, optimization algorithms are applied to further refine the calibration parameters for the correction of nonlinear lens distortions. A significant enhancement in the optimization process is achieved through the integration of the Genetic Algorithm (GA) and Particle Swarm Optimization (PSO) into a combined Integrated GA and PSO (IGAPSO) technique. The effectiveness of this method is demonstrated through the calibration of eleven roadside cameras at three different intersections. The experimental results show that when compared to the baseline DLT method, the vehicle localization error is reduced by 22.30% with GA, 22.31% with PSO, and 25.51% with IGAPSO.

## 1. Introduction

In the field of artificial intelligence, cameras play an important role as sensors for capturing visual information, enabling machines to perceive and understand their surroundings [1,2,3]. With advancements in machine vision and robot navigation [4], camera-based vision techniques have become increasingly important for extracting valuable information from visual data captured by cameras. Camera calibration [5], which is a fundamental process in computer vision, ensures precise measurements and reliable analysis by correcting distortions and estimating intrinsic and extrinsic camera parameters. Accurate camera calibration is crucial for a wide range of applications, including mobile robot navigation [6], machine vision [7], biomedical applications [8], and intelligent visual surveillance [9,10,11]. For traffic surveillance applications, calibration of roadside cameras has emerged as an active research topic. Once a roadside camera is properly calibrated, the camera can be used for vehicle localization [12], vehicle tracking [13], and sensor fusion for efficient traffic signal controls [14].

Camera calibration methods can be classified into three approaches: direct calibration methods, self-calibration methods, and calibration based on active vision. First, Direct Linear Transformation (DLT) is a widely used traditional calibration method [15]. The DLT establishes a relationship between points in the physical world and their corresponding points in the captured images. This approach is notable for its simplicity and limited number of parameters that can be easily calculated. However, it does not consider the presence of nonlinear distortion issues. Second, self-calibration methods leverage image properties to enable a camera to calibrate itself without the need for external objects [16]. This approach is especially useful in situations where manual calibration is challenging or impractical, such as in virtual reality. However, it might be less accurate than traditional calibration methods. Finally, active vision-based calibration offers an alternative to the labor-intensive process of traditional methods by enabling linear solving and ensuring robustness [17]. However, its applicability might be limited in the case of unknown or unpredictable scenarios.

To overcome not only the limitations of the traditional methods, which often neglect nonlinear distortion issues, but also to achieve higher accuracy than both self-calibration and active vision-based calibration methods, another camera calibration method utilizing graphic templates has been proposed [18]. This method utilizes the orthogonal condition of the rotation matrix and employs nonlinear optimization techniques. Although straightforward and adaptable, this method requires multiple planar template images captured from various perspectives to use as the calibration reference object. However, these template images cannot be obtained by fixed roadside cameras in traffic applications. To alleviate the need for advanced equipment in typical traffic scenarios while maintaining accuracy levels comparable to traditional methods, it is crucial to consider the effects of lens distortion. Accordingly, the current study aims to develop a method that maintains accuracy and effectively mitigates nonlinear distortion through the application of optimization algorithms. In the field of camera calibration, especially within the context of traffic surveillance and related applications, Genetic Algorithms (GAs) and Particle Swarm Optimization (PSO) are widely used due to their distinct advantages. These include their rapid convergence rates, robustness against being trapped in local optima, and proficiency in navigating the intricate, nonlinear optimization landscapes typical of camera calibration tasks. Based on these facts, GA and PSO are applied first to refine the camera calibration parameters in this paper. Then, to develop a robust optimization methodology that leverages the strengths of both methods, these two methods are ingeniously combined to form a new Integrated GA and PSO (IGAPSO) method.

The contribution of this paper is twofold. Firstly, it simplifies the transformation matrices for camera calibration, specifically tailored for traffic applications. Secondly, it introduces a novel integrated optimization algorithm, IGAPSO, aimed at enhancing the performance of both GA and PSO.

The rest of the paper is organized as follows: Section 2 reviews the relevant literature; then, the principles of traditional camera calibration are presented in Section 3; Section 4 describes the application of GA, PSO, and IGAPSO methods for camera calibration; subsequently, Section 5 provides validation results obtained from eleven traffic cameras located at three consecutive intersections; finally, Section 6 concludes the paper with an in-depth discussion.

## 2. Related Work

To address the nonlinear distortion issue, a two-step calibration process was proposed [19]. In the first step, the internal and external parameters of a camera model are determined by establishing and solving linear equations. In the second step, optimization methods are employed to refine the parameters utilizing the previously obtained parameters as initial values. Although this approach effectively addresses the distortion problem enhancing the calibration accuracy, it presents a couple of issues. First, although the traditional method can be simplified by using a limited number of parameters, it still requires the construction of three distinct matrices: the intrinsic, rotation, and translation matrices. To accurately calculate all the parameters within these matrices, a complex process of calculation and derivation is required, which is not ideal for simplified camera calibration. Second, nonlinear optimization methods, such as the gradient descent algorithm [20], can be intricate and time-consuming. If the iterative nature of the process is not well-suited, the optimization process can become unstable, leading to an inaccurate result. Thus, the key challenges lie in simplifying the calibration process and selecting the most effective optimization method.

Most studies aimed at enhancing camera calibration through optimization focus predominantly on optimizing the internal and external parameters in the intrinsic, rotation, and translation matrices [21,22]. To further simplify the calibration procedure, a more effective strategy would involve directly optimizing the parameters in the ultimate transformation matrix, which encompasses those three matrices. This approach is straightforward and integrates well with applications involving traffic cameras. In recent years, various optimization algorithms have been widely used for camera calibration [23,24]. While effective at finding local minima, gradient-based methods can be inefficient and slow when searching for the global minimum. If the initial estimate is not close to the optimal values, the optimization process may become unstable or get trapped in a local minimum. To mitigate this issue, intelligent global optimization algorithms, such as GA [25,26] and PSO [27,28], have been applied to achieve accurate camera calibration. While these methods have been successfully applied in other research fields, such as electromagnetics [29,30,31] and medicine [32], they have also shown promise in the area of camera calibration. The GA is inspired by the process of natural selection and genetics, employing genetic operators such as selection, crossover, and mutation to evolve better solutions over generations. On the other hand, PSO is based on the social behavior of flocking birds, with the position of the population adjusted based on the best positions found by individual members and by the population as a whole.

In order to effectively address multimodal problems and leverage the unique strengths of both GA and PSO, a hybrid technique known as Hybrid Genetic Algorithm and Particle Swarm Optimization (HGAPSO) has been introduced [33]. This approach aims to improve the diversity of solutions by incorporating GA’s crossover and mutation operations alongside PSO’s optimization mechanism. However, it is essential to acknowledge that the hybridization process can be complex, particularly when applied to various engineering applications. In an effort to simplify this hybridization process while maintaining a high level of optimization performance, a novel algorithm termed Integrated Genetic Algorithm and Particle Swarm Optimization (IGAPSO) is proposed in this study by combining the benefits of both GA and PSO. By introducing random mutations to all solution particles, this integrated approach aims to enhance global search capabilities and speed up convergence. The paper compares the performance of the three optimization algorithms, GA, PSO, and IGAPSO, in camera calibration for the traffic surveillance application. Using real-world traffic data captured by eleven cameras at three consecutive intersections, these algorithms are compared for their ability to fine-tune camera calibration parameters and achieve the most accurate results.

## 3. Camera Calibration

Camera-based traffic monitoring requires determining vehicle locations in world coordinates using coordinate transformations. This section provides a summary of the process involved in camera coordinate transformation.

### 3.1. Coordinate Transformation

**Figure 1:**
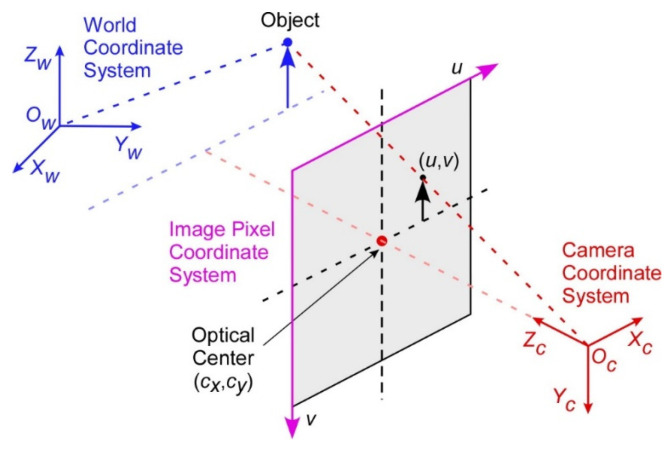
2D image, 3D camera, and 3D world coordinate systems used in the camera calibration.

The goal of camera calibration is to establish an accurate correspondence between the 2D coordinates in an image and their respective 3D coordinates in the real world. To achieve this goal, three distinct coordinate systems are utilized: the 2D image, 3D camera, and 3D world coordinate systems, as depicted in Figure 1.

The 3D world coordinates can be converted to 3D camera coordinates through the utilization of an extrinsic matrix, $M_{ex}$ based on the following relationship:

(1) $$\left[\begin{array}{c} x_{c}\\ y_{c}\\ z_{c} \end{array}\right]=M_{ex}\left[\begin{array}{c} \begin{array}{c} x_{w}\\ y_{w}\\ z_{w} \end{array}\\ 1 \end{array}\right],$$

where $\left(x_{c},\;y_{c},\;z_{c}\right)$ and $\left(x_{w},\;y_{w},\;z_{w}\right)$ represent the 3D camera coordinates and the 3D world coordinates, respectively. The 3D camera coordinates can be transformed into the 2D image coordinates using an intrinsic matrix, $M_{in}$ as shown in Equation (2):

(2) $$s\left[\begin{array}{c} u\\ v\\ 1 \end{array}\right]=M_{in}\left[\begin{array}{c} x_{c}\\ y_{c}\\ z_{c} \end{array}\right],$$

where $\left(u,v\right)$ is a pixel location in the 2D image coordinate system and s is a scaling factor.

Finally, the transformation between the 2D image pixel coordinates and the 3D world coordinates can be obtained by combining Equations (1) and (2) as:

(3) $$s\left[\begin{array}{c} u\\ v\\ 1 \end{array}\right]=P\left[\begin{array}{c} \begin{array}{c} x_{w}\\ y_{w}\\ z_{w} \end{array}\\ 1 \end{array}\right],$$

where the 3 × 4 transformation matrix P is defined by:

(4) $$P=M_{in}M_{ex}.$$

### 3.2. Camera Parameters

The intrinsic matrix, $M_{in}$, transforms the 3D camera coordinates into 2D image coordinates. The intrinsic parameters represent the camera’s optical and geometrical characteristics, which include the image center, focal length, radial lens distortion, and others. The mathematical model for the camera’s internal parameters is represented by a 3 × 3 matrix:

(5) $$M_{in}=\left[\begin{array}{ccc} f_{x} & \gamma & c_{x}\\ 0 & f_{y} & c_{y}\\ 0 & 0 & 1 \end{array}\right],$$

where $f_{x}$ and $f_{y}$ denote the focal lengths, γ represents the axis skew, and $\left(c_{x},\;c_{y}\right)$ specifies the center of the image coordinate system. For the extrinsic matrix, $M_{ex}$, the 3D world coordinates and the camera coordinates are related by a rotation matrix, R, and a 3-element translation vector, T, as:

(6) $$M_{ex}=\left[\begin{array}{cc} R_{3\times 3} & T_{3\times 1} \end{array}\right]=\left[R_{o}|T\right].$$

Based on Equations (5) and (6), the transformation matrix P in Equation (4) can be rewritten as:

(7) $$P=\left[\begin{array}{ccc} f_{x} & \gamma & c_{x}\\ 0 & f_{y} & c_{y}\\ 0 & 0 & 1 \end{array}\right]\left[R_{o}|T\right]=\left[\begin{array}{cccc} p_{1} & p_{2} & p_{3} & p_{4}\\ p_{5} & p_{6} & p_{7} & p_{8}\\ p_{9} & p_{10} & p_{11} & p_{12} \end{array}\right],$$

where $p_{i}$s represent the parameters in the resultant transformation matrix following the multiplication of the intrinsic and extrinsic matrices. Using $p_{i}$s, Equation (3) can be rewritten as:

(8) $$s\left[\begin{array}{c} u\\ v\\ 1 \end{array}\right]=\left[\begin{array}{cccc} p_{1} & p_{2} & p_{3} & p_{4}\\ p_{5} & p_{6} & p_{7} & p_{8}\\ p_{9} & p_{10} & p_{11} & p_{12} \end{array}\right]\left[\begin{array}{c} \begin{array}{c} x_{w}\\ y_{w}\\ z_{w} \end{array}\\ 1 \end{array}\right].$$

Expanding Equation (8) yields the following three linear equations:

(9) $$\left\{\begin{array}{c} u=\left(p_{1}x_{w}+p_{2}\;y_{w}+p_{3}z_{w}+p_{4}\right)/s\\ v=\left(p_{5}x_{w}+p_{6}\;y_{w}+p_{7}z_{w}+p_{8}\right)/s\\ s=p_{9}x_{w}+p_{10\;}y_{w}+p_{11}z_{w}+p_{12} \end{array}\right..$$

For the current application of traffic surveillance, all target objects in the world coordinates lie on the road, which simplifies the formulation with $z_{w}=0$. Consequently, the parameters, $p_{3}$, $p_{7}$, and $p_{11}$, are omitted from Equation (8), resulting in the following equation with a 3 × 3 matrix:

(10) $$s\left[\begin{array}{c} u\\ v\\ 1 \end{array}\right]=\left[\begin{array}{ccc} p_{11} & p_{12} & p_{13}\\ p_{21} & p_{22} & p_{23}\\ p_{31} & p_{32} & p_{33} \end{array}\right]\left[\begin{array}{c} \begin{array}{c} x_{w}\\ y_{w} \end{array}\\ 1 \end{array}\right],$$

where $p_{ij}$ represents the element in the i^th^ row and j^th^ column. To solve for the parameters in the transformation matrix, a set of homogeneous linear equations are set up and then solved by using the Singular Value Decomposition (SVD) method [34], as detailed in a prior work [12].

## 4. Optimization Algorithms

The camera calibration parameters, obtained by solving the homogeneous equations, do not adequately account for the optical distortion of the camera. Refining these parameters further through an optimization process will enhance the accuracy of coordinate transformations and minimize errors caused by optical distortion. In the field of camera calibration, various nonlinear optimization methods have been employed to achieve this, yielding improved accuracy. In this study, two commonly used global optimization algorithms, Genetic Algorithm and Particle Swarm Optimization, are used along with a newly developed integrated optimization algorithm based on the two former algorithms. These three algorithms are detailed in this section.

### 4.1. Genetic Algorithm

**Figure 2:**
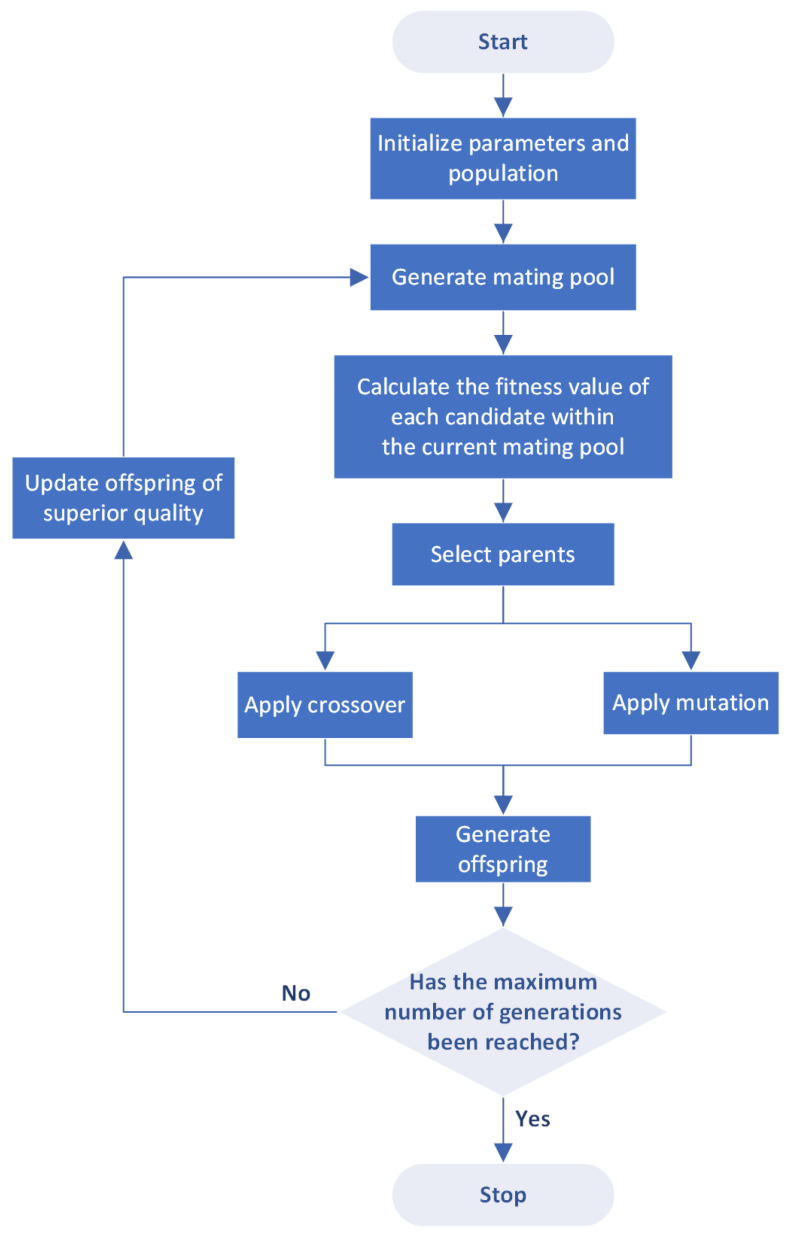
Genetic Algorithm (GA) flowchart.

The GA is a stochastic optimization algorithm inspired by Darwin’s theory of evolution. It operates on a population composed of a set of solutions, where the population size corresponds to the number of solutions. Each solution in the GA consists of a set of genes, with each gene representing a parameter in the coordinate transformation matrix for the current application. A fitness function evaluates the quality of these solutions, determining the optimal candidates. During the selection process, superior solutions are identified to form the mating pool, from which parent solutions are chosen. Selected pairs of parents from this pool produce two offspring. This pairing of high-quality parents is expected to yield offspring potentially superior to their predecessors. The reproductive process, where offspring inherit genes from their parents, is known as crossover. However, crossover alone may regenerate existing limitations of the parents by not introducing new genetic material. To address this issue, some genes undergo random alterations in a process known as mutation. This can result in offspring of superior quality, who may then replace some parents in the mating pool, influencing the subsequent generation. Figure 2 provides a flowchart detailing these steps of the GA.

Since the focus of this research is on localizing vehicles on the road surface, imposing $z_{w}=0$ leads to nine parameters, $p_{11}-p_{33}$, to be optimized in the transformation matrix. Accordingly, the configuration of the GA assigns nine genes per solution. Through multiple experimental iterations, considering both model size and operational speed, it has been determined that a population size of one hundred solutions is optimal. During the mating process, mutation occurs randomly at a rate of 30%.

### 4.2. Particle Swarm Optimization

**Figure 3:**
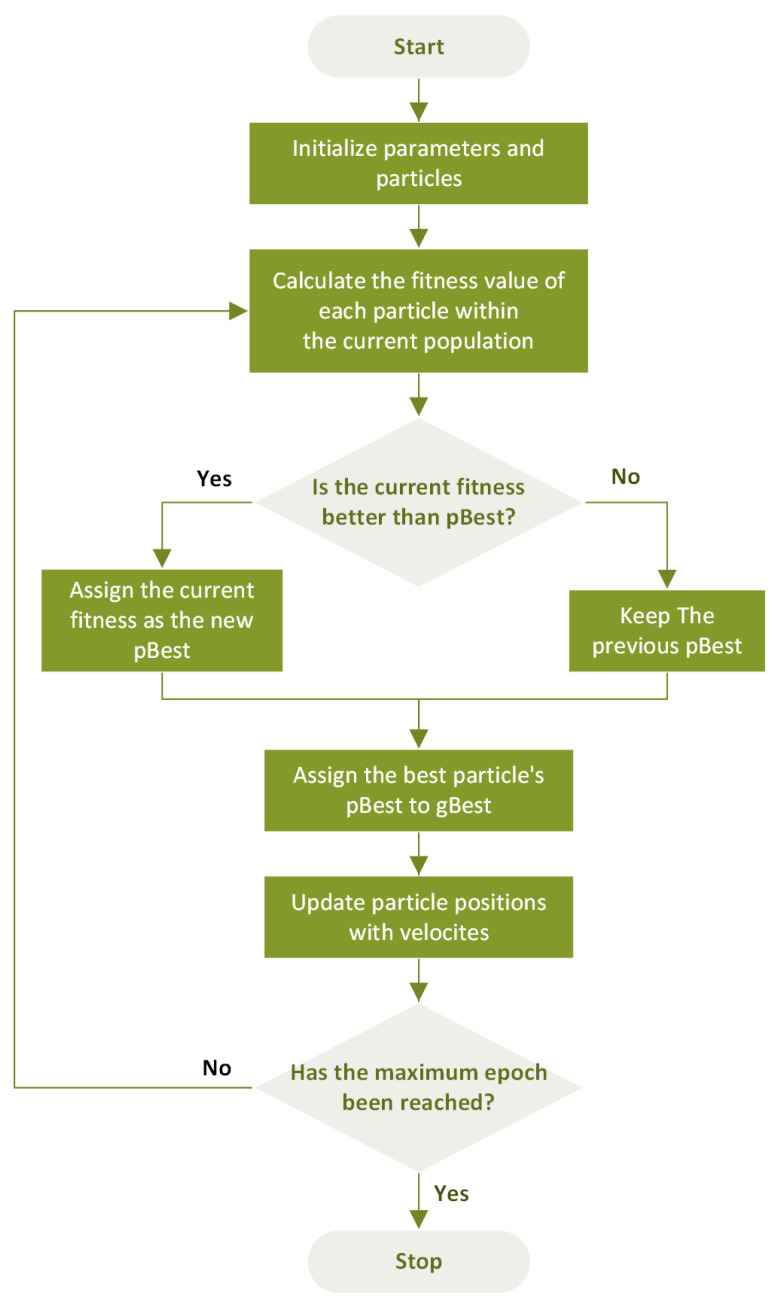
Particle Swarm Optimization (PSO) flowchart.

The PSO allows for the fast exploration of the search space and often exhibits computational efficiency across a wide range of optimization problems. In the PSO framework, a set of solutions, represented by particles, traverses the search space. They refine their positions by considering both their best individual positions and the best position found by the entire population. Each particle moves with a velocity that enables position updating over iterations to find the global minimum. The equations for updating the velocity, $V_{i}^{t}$, and position, $P_{i}^{t}$, of the ith particle at time t, can be expressed as follows:

(11) $$V_{i}^{t+1}=wV_{i}^{t}+c_{1}r_{1}\left(P_{pbest\left(i\right)}^{t}-P_{i}^{t}\right)+c_{2}r_{2}\left(P_{gbest}^{t}-P_{i}^{t}\right),$$

(12) $$P_{i}^{t+1}=P_{i}^{t}+V_{i}^{t}\Delta t,$$

where w is the inertia weight balancing between the exploration and exploitation of the best solutions found so far. $r_{1}$ and $r_{2}$ are stochastic weights, representing unique values for each particle and iteration, and $c_{1}$ and $c_{2}$ are acceleration weights, adjusting the impacts of the best individual solution and global solutions, respectively. Also, $P_{pbest}^{t}$ and $P_{gbest}^{t}$ represent the best individual position and the best global position at time t, respectively. Figure 3 shows a flowchart that outlines the steps involved in the PSO.

According to the literature, a set of benchmarks has been established to determine standard control parameters for PSO [35,36]. Notably, the best static parameters are determined to be w=0.72984 and $c_{1}+c_{2}\geq 4$. Based on these principles and various experiments, the parameters are adjusted so that $c_{2}$ increases linearly from 0.5 to 3.5 while $c_{1}$ decreases from 3.5 to 0.5 to ensure $c_{1}+c_{2}=4$. Simultaneously, w is initialized with 0.8 and gradually decreases to 0.4.

### 4.3. Integrated Algorithm Based on Genetic and Particle Swarm Optimization

In this paper, the proposed integrated algorithm starts with the PSO phase, where a swarm of particles systematically explores the search space, each adjusting its trajectory based on the best individual and global positions. This stage sets the foundation for initial solution discovery, emphasizing rapid coverage of the search domain to identify promising regions. Following the PSO phase, the proposed algorithm transitions to leveraging GA’s evolutionary strategies, introducing a selective process where solutions undergo genetic operators, crossover, and mutation. Crossover combines features from pairs of solutions, while mutation introduces slight, random changes, simulating the evolutionary concept of variation. This dual mechanism enables a diversified search beyond the initial PSO findings, aiming to refine solution quality by probing previously untouched areas within the search space.

**Figure 4:**
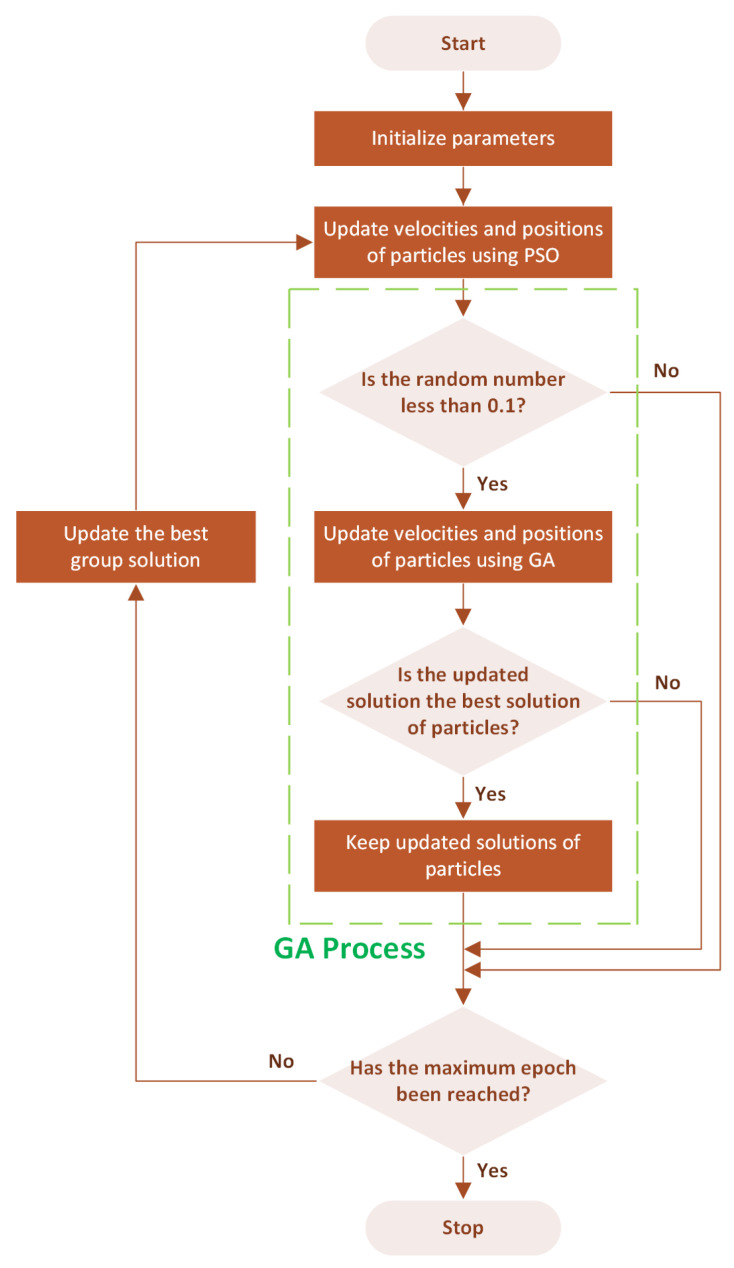
Integrated Genetic Algorithm (GA) and Particle Swarm Optimization (PSO).

During the local search procedure, if the particles find better solutions through GA-inspired operations, these improved solutions are retained, ensuring the algorithm continuously improves towards the optimal solutions. This integration of GA’s evolutionary strategies with PSO’s social behavior is termed IGAPSO. Therefore, the proposed algorithm can enhance search efficiency and solution accuracy, presenting a robust framework for complex optimization challenges such as those encountered in camera calibration. The proposed algorithm is detailed in Figure 4.

To achieve optimal results while maintaining computational efficiency, systematic experiments were conducted to tune some key parameters. Specifically, the GA was applied with a 10% probability to refine the particles, generating offspring over just ten generations. This approach effectively increased result accuracy from 0% to 10%. Further adjustments, increasing the probability to 20%, did not significantly change the outcomes. Additionally, incorporating GA into the local search process of the PSO method enhanced the quality of particles with minimal time and computational resource usage. The decision to limit GA to a maximum of ten generations was based on the observation that extending it to twenty or more generations did not substantially improve accuracy but notably increased the duration of the optimization.

## 5. Experiments

To evaluate the performance of the three different optimization algorithms for camera calibration, a total of 151 data points were collected with 3D world coordinates and their corresponding 2D image coordinates. Among these data points, 101 were used to calculate the transformation matrix, while the remaining 50 were used to assess the performance of the optimization algorithms. Based on previous research [12], the position of the camera, affixed to the traffic light pole, is designated as the origin of the 3D world coordinate system.

### 5.1. Experimental Setup

**Figure 5:**
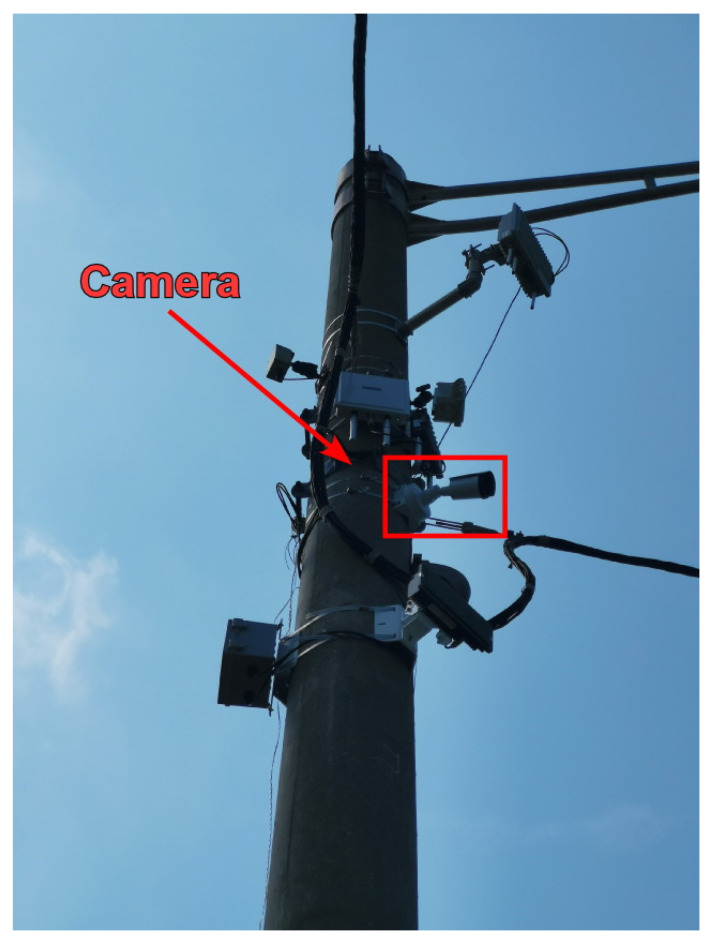
One of the network cameras installed on a traffic pole.

A network camera with a resolution of 1920 × 1080 pixels was used to capture images as shown in Figure 5. Also, to test the robustness of the proposed algorithm across various traffic surveillance scenarios, the data points were acquired from eleven different cameras, installed at various angles across three consecutive traffic intersections. To ensure the precision of the measurement points as a reference in world coordinates, a high-precision Differential Global Positioning System (DGPS) was utilized. The DGPS is capable of achieving centimeter-level accuracy in the Real-Time Kinematics (RTK) mode.

### 5.2. Experiment Using a Single Traffic Camera

**Figure 6:**
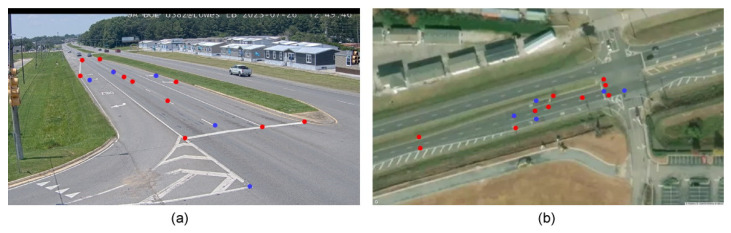
Data points collected by a single roadside camera for camera calibration. The red dots are used for the transformation matrix calculation, while the blue dots are used for validation purposes. (**a**) Data points shown on an image plane. (**b**) The corresponding data points on a Google Map.

To assess the effectiveness of the proposed integrated optimization algorithm, IGAPSO, compared to the two baseline algorithms, GA and PSO, the performances of the three algorithms were evaluated based on physical data points with DGPS reference. The data points for calibration and validation, obtained from a roadside camera, are shown in Figure 6.

Using the selected roadside camera, 10 data points (red) are obtained in a traffic scene to calculate the transformation matrix, and an additional 5 data points (blue) are chosen to test the optimization algorithms. The transformation matrix obtained by the DLT method is as follows:

(13) $$P_{DLT}=\left[\begin{array}{rrr} -0.0057 & -0.1211 & 0.1147\\ -0.0220 & 0.0082 & -0.9686\\ 0.0521 & -0.0028 & -0.1750 \end{array}\right].$$

The parameters in the transformation matrix calculated by the DLT method serve as the initial values for the optimization algorithms. When further refined using the GA, PSO, and IGAPSO algorithms, the initial transformation matrix yields results shown in Equations (14)–(16). It can be seen that the three resulting matrices contain very similar values, with an average difference of 1.3%, except for the parameter in the third row and the second column, which exhibits a maximum difference of 118% due to their relatively small values.

(14) $$P_{GA}=\left[\begin{array}{rrr} -0.0057 & -0.1216 & 0.1312\\ -0.0224 & 0.0085 & -0.9755\\ 0.0542 & 0.0005 & -0.1048 \end{array}\right],$$

(15) $$P_{PSO}=\left[\begin{array}{rrr} -0.0056 & -0.1203 & 0.1302\\ -0.0223 & 0.0085 & -0.9718\\ 0.0537 & -0.0002 & -0.1041 \end{array}\right],$$

(16) $$P_{CGAPSO}=\left[\begin{array}{rrr} -0.0057 & -0.1222 & 0.1324\\ -0.0224 & 0.0084 & -0.9761\\ 0.0543 & 0.0011 & -0.1067 \end{array}\right].$$

**Figure 7:**
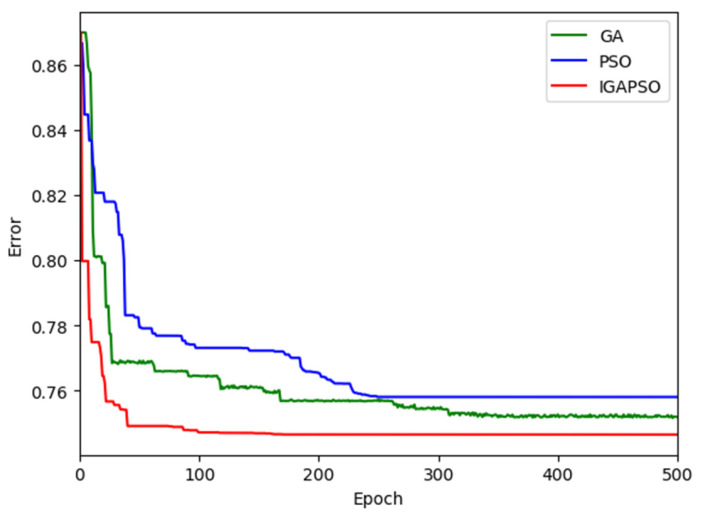
Comparison of convergence rates of GA, PSO, and IGAPSO for the corresponding traffic scenes shown in Figure 6.

The camera calibration errors, calculated as the average Euclidean distances between the world coordinates of the data points obtained by the DGPS and those obtained by the calibrated camera, are presented in Table 1. For these calculations, the world coordinates obtained by the DGPS are regarded as the ground truth against which the coordinates determined by the different algorithms are compared. In the table, it is shown that the average error obtained by the initial DLT is 0.87 m, while the three optimization algorithms further reduce the error. Specifically, the GA, PSO, and IGAPSO show improvements of 13.8%, 12.6%, and 14.9% over the baseline DLT method, respectively. Additionally, the proposed integrated optimization algorithm exhibits a faster convergence rate compared to the other two optimization algorithms, as shown in Figure 7.

### 5.3. Experiment Using Multiple Traffic Cameras

**Figure 8:**
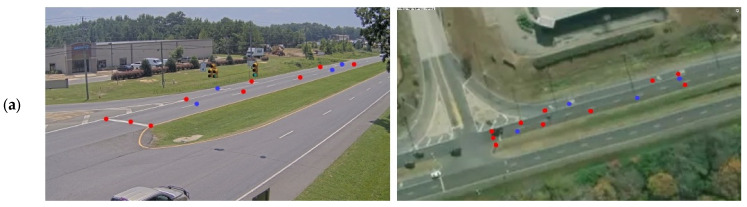

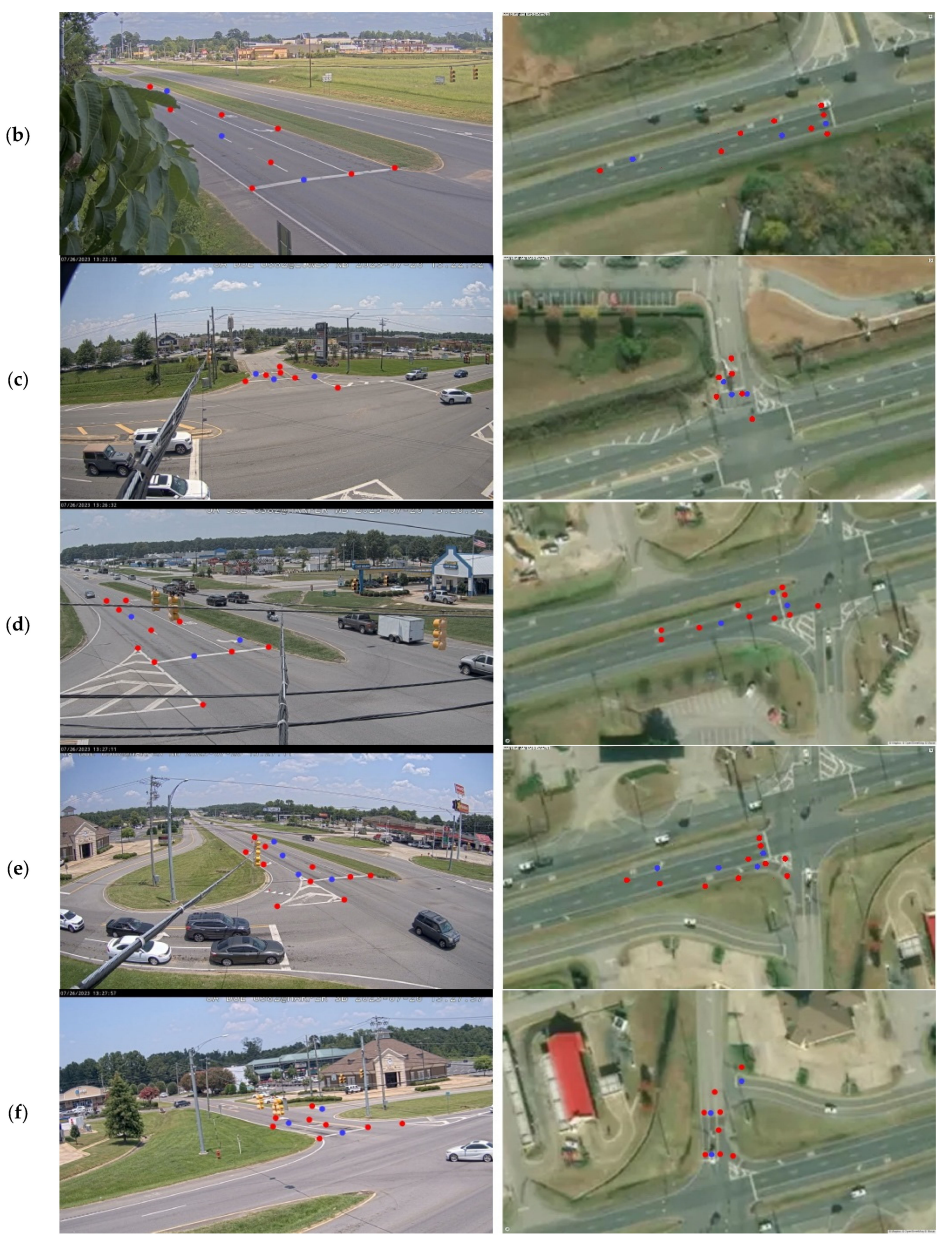
Subfigures (**a**–**f**) display data points from images captured by 6 different roadside traffic cameras on the left, alongside corresponding visualizations on Google Maps on the right, illustrating a variety of traffic scenes.

In this study, a total of eleven roadside cameras are employed to capture multiple traffic scenes from various viewing angles at three consecutive intersections. The traffic scene presented in Section 5.2 serves as an illustrative example, captured by one of the cameras, to demonstrate the performance of three distinct optimization algorithms utilized for camera calibration. The dataset collected from the remaining ten cameras comprises a total of 136 data points, with 96 of these points being used for the calculation of the transformation matrix. The remaining 40 data points were allocated for testing the performance of the three optimization algorithms. Aside from the scenario detailed in Section 5.2, Figure 8 illustrates six out of ten traffic scenes, each depicting a unique scenario. In the figure, red and blue dots represent data points used for the calculation of the transformation matrix and for validating the results of the optimization algorithms, respectively. The images on the left show the data points in the 2D camera image plane, while those on the right present the corresponding data points on Google Maps.

**Figure 9:**
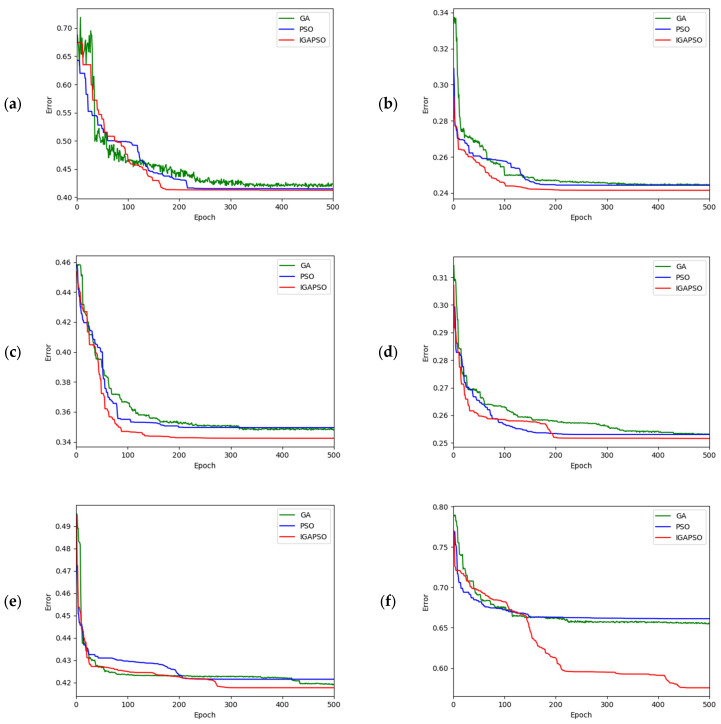
Subfigures (**a**–**f**) show convergence performances of three different optimization algorithms for the corresponding traffic scenes shown in Figure 8a–f.

The convergence performances of the three different optimization algorithms are graphically presented in Figure 9 for the corresponding six different traffic scenes shown in Figure 8. In these figures, the green, blue, and red curves represent the calibration errors for the GA, PSO, and IGAPSO algorithms, respectively. Due to the stochastic nature of optimization algorithms, the outcomes can vary with each execution, leading to different convergence trajectories and potentially sub-optimal results in some instances. To mitigate this variability, each algorithm is executed five times, and the results are averaged across these runs. This approach helps mitigate the impact of inherent randomness in stochastic optimization processes, yielding a more reliable and compelling set of results. As shown in Figure 9, the proposed integrated optimization algorithm consistently exhibits faster convergence rates compared to the other two conventional optimization methods in most cases.

In Table 2, the camera calibration errors, calculated as the average Euclidean distances between the world coordinates of the data points obtained by the DGPS and those obtained by the camera calibration, are presented. The optimization results for certain cameras show significant enhancements, while others exhibit only modest improvements. This disparity can be attributed to varying degrees of distortion in each camera. Overall, the optimization-based refinement resulted in substantial improvements in average performance: 22.30% with GA, 22.31% with PSO, and 25.51% with IGAPSO.

**Figure 10:**
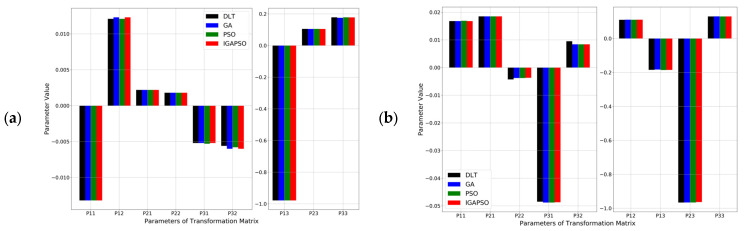

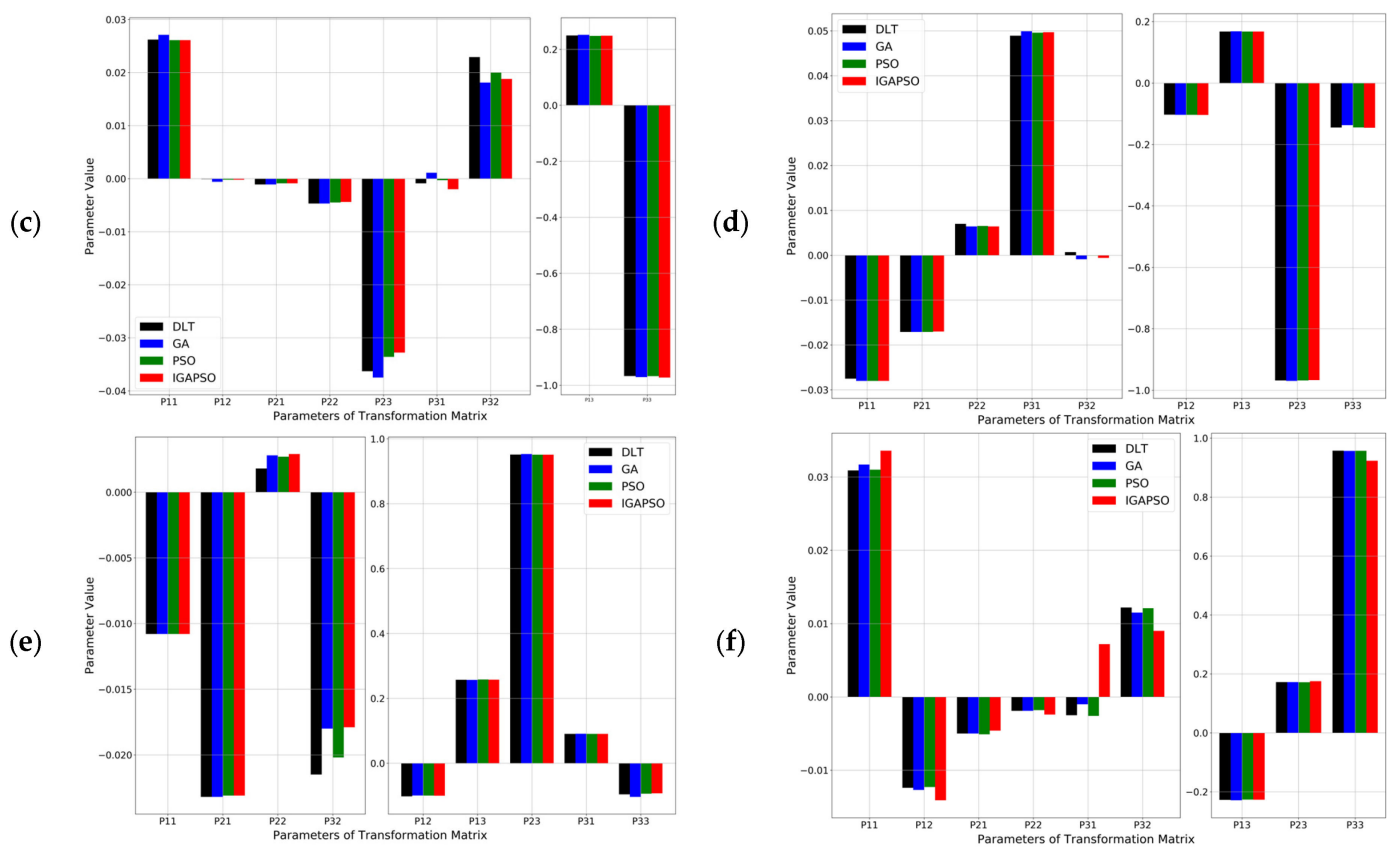
Bar plot representation of the nine parameters comprising the 3 × 3 transformation matrix, denoted as $P_{ij}$ where i represents the row and j represents the column. Subfigures (**a**–**f**), corresponding to Figure 8a–f and Figure 9a–f, illustrate the results obtained from DLT and three other algorithms, each represented by a distinct color bar. The horizontal axes denote the parameters $P_{ij}$, while the vertical axes show their corresponding values.

As explained in Section 3, the original camera coordinate transformation matrix of 12 parameters is reduced to a 3 × 3 matrix of 9 parameters for the traffic surveillance application by ignoring the vertical coordinate of the data points. To ensure the robustness of the proposed approach in diverse traffic scenarios, 151 data points were collected using eleven different roadside cameras offering various view angles to the traffic scenes. The resulting matrix parameters for the six cases depicted in Figure 8 and Figure 9, labeled (a) to (f), are presented as bar graphs in Figure 10. In the figure, the transformation matrix parameters for DLT and three different optimization algorithms are presented for each of the six cases. Due to the large disparity in the magnitude of the values, two vertical axes are used for each plot. The nonlinear optimization algorithms refine the transformation matrix parameters obtained by DLT to rectify distortion issues stemming from uncertain internal and external camera calibration parameters. Consequently, although the differences in individual parameters for the same camera may seem minor, modifying the parameters of the simplified transformation matrix leads to noticeable improvements in the vehicle localization.

Note that the presented method for refining the transformation matrix through optimization requires ground truth data, which is typically obtained using advanced equipment such as a DGPS. In situations where acquiring ground truth data is challenging or impractical, QR decomposition, as demonstrated in the authors’ previous work [12], offers a viable method for estimating the ground truth data without the need for advanced equipment. The approach provides a convenient and computationally efficient means of determining the world coordinates of locations with an acceptable level of error.

## 6. Conclusions

This paper presents an optimization-based camera calibration approach for accurately determining the 3D world coordinates of ground points, a crucial factor for accurate vehicle localization in traffic monitoring applications. Initially, the conventional Direct Linear Transform (DLT) method is utilized to compute the coordinate transformation matrix using 151 data points, collected using eleven different roadside cameras. Subsequently, using the results of the DLT method as initial parameters, three different optimization algorithms—the Genetic Algorithm (GA), Particle Swarm Optimization (PSO), and the newly proposed Integrated Genetic Algorithm-Particle Swarm Optimization (IGAPSO)—are applied to further refine the transformation matrix. IGAPSO leverages the advantages of GA and PSO to enhance both the convergence rate and optimization performance. The optimization-based approach shows a significant reduction in the average localization errors by 22.30% with GA, 22.31% with PSO, and 25.51% with IGAPSO compared to the baseline DLT method. Among these, IGAPSO not only demonstrated superior results compared to GA and PSO, but also exhibited faster convergence in most cases.

This study presented a refinement process for the transformation matrix by applying optimization algorithms, bypassing the need for intricate geometric models and complex mathematical derivations associated with internal and external camera parameters. By evaluating the proposed algorithm using data collected from eleven roadside cameras capturing diverse angles at three consecutive intersections, the robustness of the approach has been demonstrated across a wider range of traffic scenarios. The introduction of a more efficient and effective roadside camera calibration method significantly contributes to advancements in the field of traffic surveillance for data analysis and control purposes.

Although it has successfully demonstrated the effectiveness of the proposed approach, there are limitations in this study. This study relies on data collected from eleven roadside cameras capturing diverse angles at three consecutive intersections, which may limit the generalizability of the findings to other environments or conditions. Additionally, while the proposed IGAPSO algorithm shows promising results, further comprehensive evaluation across a broader range of datasets and scenarios is necessary to validate its effectiveness in diverse real-world applications. Moreover, the simplifications and assumptions made in the optimization-based approach may affect the accuracy and applicability of the results in certain scenarios.

For future work, extending data collection efforts to include larger and more diverse datasets from various traffic environments would be beneficial to validate the performance and robustness of the proposed algorithm under different conditions. Additionally, continual refinement and optimization of the IGAPSO algorithm could improve its efficiency, accuracy, and scalability, possibly through hybridization with other optimization techniques or the incorporation of adaptive strategies. Furthermore, conducting field tests and the real-world deployment of the optimized camera calibration approach would be essential to assess its performance and feasibility in practical traffic surveillance applications, considering factors such as real-time processing, scalability, and hardware constraints.

## Figures and Tables

**Table 1:** Comparison of camera calibration errors by DLT, GA, PSO, and IGAPSO for data points obtained by a single roadside camera (best result shown in bold).

	Linear Transformation (DLT)	Genetic Algorithm (GA)	Particle Swarm Optimization (PSO)	Integrated GA and PSO (IGAPSO)
Error (improvement)	0.87 m	0.75 m (13.8%)	0.76 m (12.6%)	**0.74 m (14.9%)**

**Table 2:** Comparison of camera calibration errors by DLT, GA, PSO, and IGAPSO in all eleven distinct traffic scenes (best results shown in bold).

Camera	Calibration Errors (Performance Improvement over Baseline DLT)			
	Linear Transformation (DLT)	Genetic Algorithm (GA)	Particle Swarm Optimization (PSO)	Integrated GA and PSO (IGAPSO)
Cam 1	0.64 m	0.42 m (34.38%)	0.42 m (34.38%)	**0.41 m (35.94%)**
Cam 2	0.33 m	0.24 m (27.27%)	0.24 m (27.27%)	**0.23 m (30.33%)**
Cam 3	0.48 m	0.29 m (39.58%)	0.28 m (41.67%)	**0.27 m (43.75%)**
Cam 4	0.87 m	0.75 m (13.79%)	0.75 m (13.79%)	**0.74 m (14.94%)**
Cam 5	0.56 m	0.53 m (5.36%)	**0.52 m (7.14%)**	**0.52 m (7.14%)**
Cam 6	0.46 m	0.35 m (23.91%)	0.35 m (23.91%)	**0.34 m (26.09%)**
Cam 7	0.41 m	0.29 m (29.27%)	0.29 m (29.27%)	**0.28 m (31.71%)**
Cam 8	0.60 m	0.48 m (20.00%)	0.48 m (20.00%)	**0.47 m (21.67%)**
Cam 9	0.31 m	**0.25 m (19.35%)**	**0.25 m (19.35%)**	**0.25 m (19.35%)**
Cam 10	0.50 m	0.42 m (16.00%)	0.42 m (16.00%)	**0.41 m (18.00%)**
Cam 11	0.79 m	0.66 m (16.46%)	0.69 m (12.66%)	**0.54 m (31.65%)**

## Data Availability

No new data were created or analyzed in this study. Data sharing not applicable.
